# Decreased levels of Fibroblast Growth Factor 21 are correlated with improved hypoglycemia in patients with insulinoma

**DOI:** 10.1038/srep43123

**Published:** 2017-02-22

**Authors:** Xu Li, Haoyong Yu, Jun Yin, Lianxi Li, Jian Zhou, Ming Li, Qing Li, Haibing Chen, Fang Liu, Yuqian Bao, Junfeng Han, Weiping Jia

**Affiliations:** 1 Department of Endocrinology and Metabolism, Shanghai Jiao Tong University Affiliated Sixth People’s Hospital, Shanghai Clinical Center for Diabetes, Shanghai Diabetes Institute, Shanghai Key Laboratory of Diabetes Mellitus, Shanghai Key Clinical Center for Metabolic Disease, 600Yishan road, Shanghai, 200233 China

## Abstract

Fibroblast growth factor-21 (FGF-21) improves insulin sensitivity and lipid metabolism in obese or diabetic animal models and has been proposed as a potential therapeutic agent for treating T2DM, obesity, and their related complications. However, little is known about the changes of FGF21 levels in response to endogenous hyperinsulinemic hypoglycemia. To explore its relationship with parameters of glucose metabolism in patients with insulinoma, eleven subjects with pathological insulinoma and twenty-two healthy subjects were recruited for this study. Interestingly, we found that the serum FGF21 levels increased significantly in patients with insulinoma at baseline compared with the control group (381.36 ± 107.12 vs. 62.59 ± 10.48 pg/mL; *P* = 0.001). Furthermore, FGF21 was positively correlated with insulin (*r* = 0.80, *P* = 0.003) and proinsulin (*r* = 0.72, *P* = 0.012) in subjects with insulinoma. Multiple stepwise regression analysis showed that FGF21 was independently associated with insulin (*β* = 0.80, *P* = 0.003). In addition, FGF21 decreased significantly after surgery, and its change was still correlated positively with the changes in insulin (*r* = 0.61, *P* = 0.048) and proinsulin (*r* = 0.84, *P* = 0.001). These findings suggested that the serum FGF21 levels could be involved in a complex adaptive response to insulin secretion and glucose metabolism in humans.

Fibroblast growth factor-21 (FGF-21), a member of the FGF subfamily, has been reported to have multiple salutary metabolic effects in animals^1^,^2^,^3^. The specific over-expression of FGF21 in the livers of mice is resistant to diet-induced weight gain, whereas FGF21 administration reduces the plasma glucose and triglyceride levels to near-normal levels and improves the metabolic state of diabetic monkeys^1^,^4^. Patients with obesity and type 2 diabetes receiving LY2405319 (LY), a variant of FGF21, produced significant improvements in dyslipidemia. However, only a trend toward glucose lowering was observed^5^. Numerous studies point to FGF21 as a potential therapeutic agent for treating T2DM, obesity and their related complications^1^,^6^. In 2007, Badman and Inagaki established FGF21 as a major endocrine regulator of the response to fasting by regulating adipose tissue (lipolysis), liver (fatty acid oxidation and ketogenesis), and brain (torpor)^7^,^8^.

Can a hormone that enhances weight loss and induces thermogenesis be unregulated during starvation? The results seem controversial in different human studies^9^. FGF21 levels demonstrated a modest 74% increase after a 7-day fast in patients with rheumatoid arthritis^10^. However, women with anorexia nervosa, which is a state of chronic nutritional deprivation, have reduced or similar levels of FGF21 compared with the levels in normal-weight controls^11^,^12^. Appropriate counter-regulatory hormone responses to hypoglycemia are critical for maintaining blood glucose levels within a narrow range. Whether serum FGF21 is responsive to endogenous hyperinsulinemic hypoglycemia in humans has never been explored. In the present study, we tested this hypothesis and therefore recruited subjects with insulinoma with symptoms of hypoglycemia and followed them up after the surgery.

## Materials and Methods

### Study population

Eleven subjects with pathological insulinoma (age, 53.18 ± 3.12 years) who underwent tumorectomy in our hospital and twenty-two age- and sex-matched healthy controls (age, 50.82 ± 1.61 years) were included in the study. The diagnosis of insulinoma was based on the clinical hypoglycemia symptoms and the following four criteria: 1) documented blood glucose levels near or below 50 mg/dL (<2.8 mmol/L); 2) concomitant insulin levels equal or greater than 3 mU/L (>21 pmol/L); 3) elevated C-peptide levels (>0.6 ng/mL or >0.2 nmol/L); and 4) absence of sulfonylurea in the plasma^13^. In addition, each case underwent tumor localization by different tools, including CT, MRI and intra-arterial calcium-stimulated venous sampling (ASVS). The CT scan was usually our first choice for the location and imaging of the tumor. If negative, MRI was used. If the imaging of both tools was negative, ASVS was the final choice before surgery. Eventually, surgical pathology confirmed the diagnosis of insulinoma in each patient with insulinoma. Subjects with the following conditions were excluded from this study: biliary obstructive disease, acute or chronic virus hepatitis, cirrhosis, diabetes mellitus, and thyroid disorders. Those who had ever received insulin or thiazolidinediones were also excluded. Current drinkers and ex-drinkers were excluded from the study. Current drinkers were those who drank 6 g or more alcohol/d on average for at least 1 year, and ex-drinkers were those who had been a drinker but quit for at least half a year before the survey. Written informed consent was provided by all participants before being enrolled in the study. The study was carried out in accordance with the guidelines proposed in the Declaration of Helsinki and approved by the Ethics Committee of Shanghai Jiaotong University Affiliated Sixth People’s Hospital.

### Anthropometric and biochemical measurements

All subjects were evaluated after an overnight fast of at least ten hours. Anthropometric assessments included height, weight measurements. BMI was calculated as the weight in kilograms divided by the square of the height in meters. Fasting plasma glucose (FPG) was measured by the hexokinase method. Hemoglobin A1c (HbA1c) was measured by high performance liquid chromatography with an HLC-73G7 automated glycohemoglobin analyzer (Tosoh, Tokyo, Japan). The other biochemical indexes were measured on a Hitachi 7600 analyzer (Hitachi, Tokyo, Japan). Serum insulin was assayed using radioimmunoassay (Linco Research, St Charles, MO, USA). Serum C-peptide was assayed using radioimmunoassay (Linco Research, St Charles, MO, USA). Serum FGF21 was measured using an ELISA kit established in another laboratory^14^ (Antibody and Immunoassay Services, the University of Hong Kong, Hong Kong). In addition to determining the FGF21, proinsulin, insulin, C-peptide, and plasma glucose levels, we performed a routine blood examination and liver and renal function tests to evaluate the safety of surgery in patients with insulinoma within one week after surgery. All laboratory measurements met the Shanghai Center for Clinical Laboratory criteria.

### Statistical analysis

All statistical analyses were performed with the Statistical Package for Social Science version 21.0 (SPSS, Inc., Chicago, IL, USA). Normally distributed data were expressed as the mean ± SEM. Data that were not normally distributed, as determined using the Shapiro-Wilk test, were expressed as a median with interquartile range. Student’s unpaired *t* test or the Mann-Whitney U test was used for group comparisons, as appropriate. The correlations between FGF21 and other values were estimated by Pearson correlation and Spearman correlation analysis. Multiple regression analysis was used to show the independent relationship of FGF21 with other parameters. The levels of insulin, C-peptide and FGF21 in patients with insulinoma before and after surgery were compared using the paired *t* test or Wilcoxon signed rank test as appropriate. All *P* values <0.05 denoted statistical significance.

## Results

### Characteristics of the insulinoma and normal control groups

The general and biochemical characteristics of the patients with insulinoma (5 males and 6 females) and normal controls (11 males and 11 females) are shown in Table 1. Aspartate aminotransferase (AST), *γ*-glutamyltranspeptidase (*γ*-GT), alkaline phosphatase (ALP), total cholesterol (TC) and triglycerides (TG), low-density lipoprotein cholesterol (LDL), creatinine (Cr) and uric acid did not significantly differ between the groups (all *P* > 0.05). FPG [3.35 ± 0.42 vs. 4.92 (4.75–5.26) mmol/L; *P* = 0.002], HbA1c [4.50 (4.40–4.80) vs. 5.39 ± 0.07; *P* = 0.006] and high-density lipoprotein cholesterol[HDL, 1.03 ± 0.07 vs. 1.31 (1.11–1.48) mmol/L; *P* = 0.014] were significantly reduced in the insulinoma group, whereas BMI (26.59 ± 0.98 vs. 21.59 ± 0.32 kg/m^2^; *P* < 0.001), alanine aminotransferase [ALT, 34.91 ± 7.38 vs. 15.00 (10.75–20.00) U/L; *P* = 0.022], fasting insulin (30.47 ± 4.93 vs. 6.65 ± 0.74 mU/L; *P* = 0.001), fasting C-peptide (4.46 ± 0.62 vs. 1.67 ± 0.12 ng/mL; *P* = 0.001) and FGF21 (381.36 ± 107.12 vs. 62.59 ± 10.48 pg/mL; *P* = 0.001) were markedly increased in patients with insulinoma compared with sex- and age-matched healthy controls (all *P* < 0.05).

### Correlations of FGF21 with other studied characteristics in subjects with insulinoma

Previous results have shown that theFGF21 levels were increased in subjects with insulinoma. To investigate the factors associated with FGF21, we performed Pearson correlation and Spearman correlation analysis between FGF21 and other biochemical characteristics. As expected, plasma FGF21 was significantly positively correlated with ALT (*r* = 0.66, *P* = 0.011), AST (*r* = 0.73, *P* = 0.011), fasting insulin (*r* = 0.80, *P* = 0.003) and fasting proinsulin (*r* = 0.72, *P* = 0.012). We failed to find significant relationships between FGF21 and age, BMI, TG, FPG, and fasting C-peptide in patients with insulinoma (Table 2). Multiple regression analysis showed that fasting insulin (*β = *0.80, *P* = 0.003) was an independent predictor of serum FGF21 levels in subjects with insulinoma (Table 3).

### The influence of surgical resection of the tumor on plasma levels of FGF21, insulin and proinsulin in subjects with insulinoma

**Figure 1:**
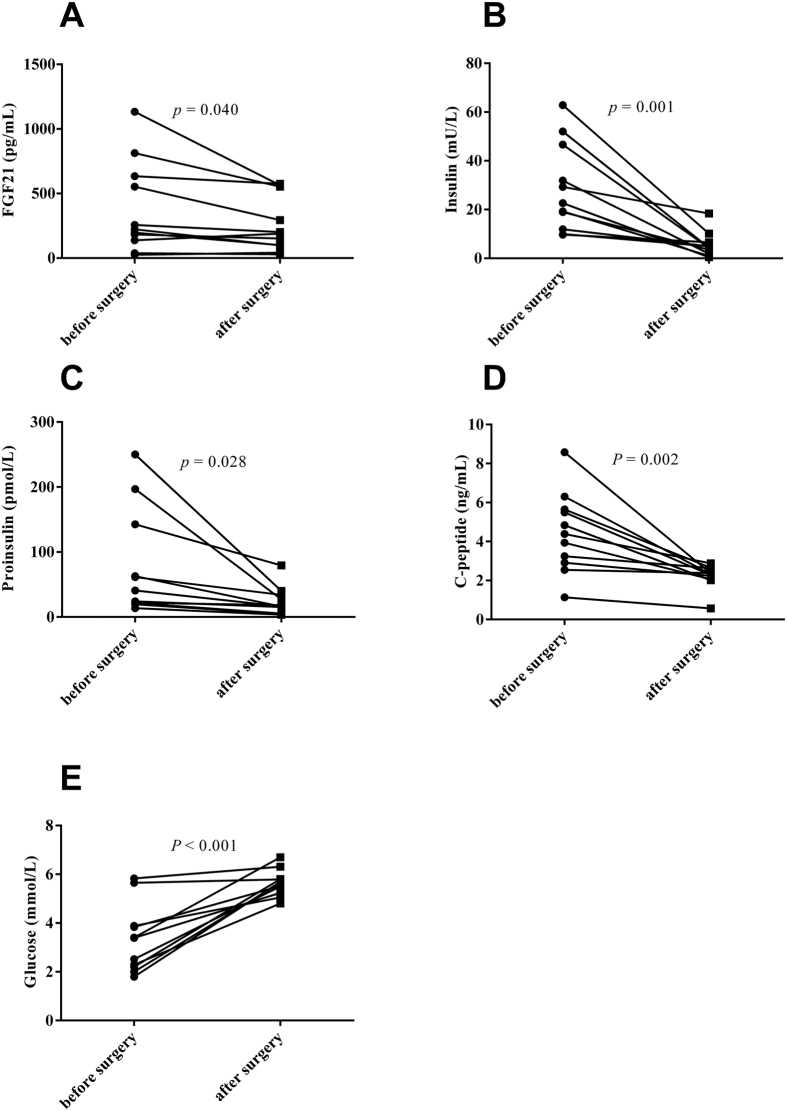
Plasma levels of FGF21 (**A**), insulin (**B**), proinsulin (**C**), C-peptide (**D**) and glucose (**E**) in patients with insulinoma before and after surgery.

**Figure 2:**
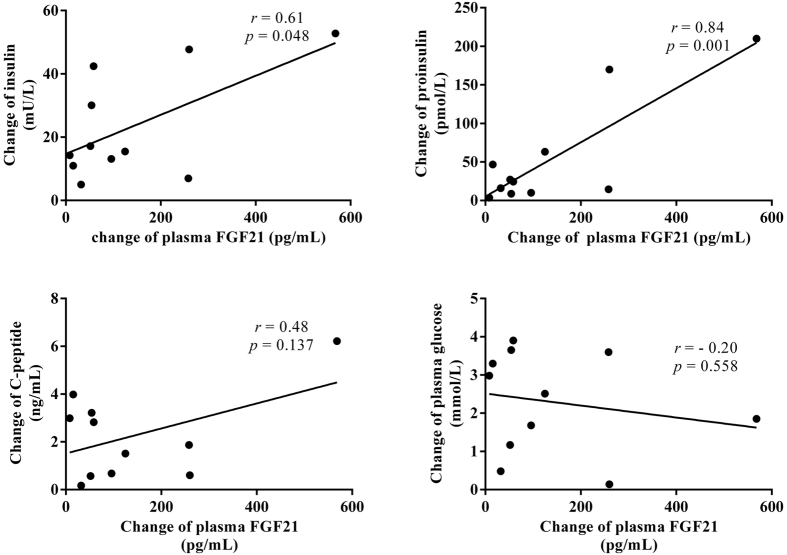
Relationship of plasma ΔFGF21 with Δinsulin (**A**), Δproinsulin (**B**), ΔC-peptide (**C**) and Δglucose (**D**) in patients with insulinoma after surgery.

Nearly two-thirds of all insulinoma patients can be cured by resection of the tumor. In our study, we found decreased insulin (before surgery, 30.47 ± 4.93 mU/L; after surgery, 6.28 ± 1.35 mU/L), proinsulin (before surgery, 77.74 ± 24.61 pmol/L; after surgery, 23.73 ± 6.60 pmol/L) and C-peptide levels (before surgery, 4.46 ± 0.62 ng/mL; after surgery, 2.22 ± 0.18 ng/mL) after surgery in all patients with insulinoma. Furthermore, the plasma concentrations of FGF21 in patients with insulinoma were significantly reduced (before surgery, 381.36 ± 107.12 pg/mL; after surgery, 254.80 ± 64.10 pg/mL) when blood glucose improved after surgery (before surgery, 3.35 ± 0.42 mmol/L; after surgery, 5.64 ± 0.16 mmol/L) (all *P* < 0.05; Fig. 1). In addition, the changes in the plasma FGF21 levels were positively correlated with the changes in insulin (*r* = 0.61, *p* = 0.048), proinsulin (*r* = 0.84, *p* = 0.001), C-peptide (*r* = 0.48, *p* = 0.137) and plasma glucose (*r* = 0.20, *p* = 0.558) in patients with insulinoma (Fig. 2).

## Discussion

Mounting evidence from animal-based studies suggests that FGF21 may be an emerging therapeutic target for type 2 diabetes mellitus^3^,^15^,^16^. The present study provides the first evidence that shows that the FGF21 concentrations were significantly increased in patients with insulinoma and decreased after removing the tumor. Its biphasic change was correlated positively with insulin and proinsulin. These findings suggest that the circulating levels of FGF21 may change in response to insulin secretion and glucose metabolism and thus provide insight into the role of FGF21 as a metabolic regulator in humans.

Consistent with our present findings, FGF21 did not induce hypoglycemia, even in fasted FGF21-transgenic mice^1^. It is of interest that previous study has also shown that FGF21 helps maintain blood glucose during prolonged fasting via the stimulation of gluconeogenesis^17^. FGF21 may participate in the late starvation response as a hormonal link between tissue stress and the liberation of amino acids and their downstream utilization by the liver for gluconeogenesis and/or the shunting of newly synthesized glucose to needy tissues. In addition, FGF21 has been found to play a role in PPARα-mediated ketone body production by increasing fatty acid oxidation^8^. FGF21 administration stimulates lipolysis and glucose uptake in adipocytes and reduces body temperature by decreasing physical activity^18^. FGF21 acts as a fasting-induced hormone in humans, which indicates that it contributes to the late stages of adaptive starvation and regulates the utilization of fuel derived from tissue breakdown^19^. Although FGF21 might be increased by metabolic stress in patients of insulinoma and decreased after the surgery, the correlation of FGF21 with fasting plasma glucose had not been found in our study.

There was a complicated crosstalk between FGF21 and insulin. In rodents, FGF21 is highly expressed in the pancreas^20^,^21^ and may act in an autocrine/paracrine manner to protect from glucolipotoxicity and cytokine-induced apoptosis^20^. In humans, fasting FGF21 levels were significantly associated with the insulin and with C-peptide levels^22^. A glucose clamp-based study in humans demonstrated that the elevation of serum insulin acutely suppresses FGF21 production^23^. Zhuofeng Lin *et al*. observed the biphasic change of serum FGF21 levels after an oral glucose challenge, which was opposite to the pattern of insulin^24^. Although the insulin levels are low during fasting and peak after feeding and FGF21 expression is regulated in the opposite manner in healthy animals^8^,^25^, it is well established that both FGF-21 mRNA and protein were induced by Akt1 overexpression or insulin stimulation in skeletal muscle cells^26^. However, in the present study, we found that increased FGF21 concentrations in patients with insulinoma and decreased significantly after tumorectomy. Nevertheless, whether the pancreatic islet is a potential source of circulating FGF21 and the interplay between insulin and FGF21 under pathological conditions needed to be further investigated in the future. They are necessary for FGF21 to be used as a novel treatment tool for metabolic diseases.

This study has several limitations, including the small population size and its cross-sectional nature, and we should bear in mind that the groups were not matched for BMI. In addition, the findings in the study were based on a special population with insulinoma. Other studies have found evidence of FGF21 release in response to other nutritional interventions, such as prolonged protein restriction^27^ and amino acid deficiency^28^,^29^, fructose ingestion^30^ and overfeeding of carbohydrates^31^. It is worth noting that the studies mentioned above only demonstrated the role of single energy substance, and the findings cannot be extrapolated to mixed meals which humans ingest every day. The food type of patients with insulioma was the same as normal to maintain the blood glucose level, and the patients were informed to do an overnight fast for at least ten hours, which may be eliminate the effects of food consumption on FGF21 levels. Additional work is required to precisely define the threshold concentration of FGF21 to drive metabolic responses.

In conclusion, a key strength of this study is the observation of dynamic changes of FGF21 in patients with insulinoma. Most interestingly, the FGF21 concentrations decreased significantly after removing the tumor and correlated positively with the levels of insulin and proinsulin. This implies that FGF21 contributes to the metabolic adaptations of the body to insulinoma. FGF21 thus holds promise as an excellent drug target for the treatment of metabolic diseases characterizing impaired glucose homeostasis.

## Additional Information

**How to cite this article**: Li, X. *et al*. Decreased levels of Fibroblast Growth Factor 21 are correlated with improved hypoglycemia in patients with insulinoma. *Sci. Rep.* **7**, 43123; doi: 10.1038/srep43123 (2017).

**Publisher's note:** Springer Nature remains neutral with regard to jurisdictional claims in published maps and institutional affiliations.

## Figures and Tables

**Table 1:** Baseline clinical and laboratory characteristics of the study population.

	Normal group (N = 22)	Insulinoma (N = 11)	
		Before surgery	After surgery
Male: Female	11:11	5:6	5:6
Age (years)	50.82 ± 1.61	53.18 ± 3.12	—
BMI (kg/m^2^)	21.59 ± 0.32	26.59 ± 0.98^**^	26.21 ± 0.94
ALT (U/L)	15.00 (10.75–20.00)	34.91 ± 7.38^*^	37.00 ± 4.48
AST (U/L)	20.14 ± 0.93	26.55 ± 3.72	33.18 ± 3.90
γ-GT (U/L)	22.86 ± 1.97	32.91 ± 7.15	34.09 ± 3.70
ALP (U/L)	71.00 (66.00–87.00)	70.00 ± 60.05	81.45 ± 9.21
TC (mmol/L)	4.47 ± 0.19	3.98 ± 0.19	—
TG (mmol/L)	1.18 ± 0.10	1.29 (1.20–1.40)	—
HDL (mmol/L)	1.31 (1.11–1.48)	1.03 ± 0.07^*^	—
LDL (mmol/L)	2.45 ± 0.15	2.57 ± 0.12	—
Cr (μmol/L)	68.00 ± 1.85	69.00 (24.59–82.00)	69.73 ± 5.65
Uric acid (μmol/L)	310.64 ± 12.62	340.82 ± 24.26	296.36 ± 19.33
FPG (mmol/L)	4.92 (4.75–5.26)	3.35 ± 0.42^**^	5.64 ± 0.16^##^
Fasting insulin (mU/L)	6.65 ± 0.74	30.47 ± 4.93^**^	6.28 ± 1.35^##^
HbA1c (%)	5.39 ± 0.07	4.50 (4.40–4.80)^**^	—
Fasting C-peptide (ng/mL)	1.67 ± 0.12	4.46 ± 0.62^**^	2.22 ± 0.18^##^
AFP (ng/mL)	5.56 ± 0.87	3.91 ± 0.88	—
CEA (ng/mL)	1.57 ± 0.33	2.43 ± 0.46	—
CA199 (U/mL)	9.16 ± 2.40	8.77 ± 1.37	—
FGF21 (pg/mL)	62.59 ± 10.48	381.36 ± 107.12^**^	254.80 ± 64.10^#^
Proinsulin (pmol/L)	—	77.74 ± 24.61	23.73 ± 6.60^#^

^*^P < 0.05, ^**^P < 0.01insulinoma (before surgery) vs. normal group.

^#^*P* < 0.05, ^##^*P* < 0.01 insulinoma (before surgery) vs. insulinoma (after surgery).

Normally distributed data were expressed as mean ± SEM. Data that were not normally distributed were expressed as median with interquartile range.

Abbreviations: BMI, body mass index; ALT, alanine aminotransferase; AST, aspartate aminotransferase; *γ*-GT, *γ*-glutamyltranspeptidase; ALP, alkaline phosphatase; TC, total cholesterol; TG, triglycerides; HDL, high-density lipoprotein cholesterol; LDL, low-density lipoprotein cholesterol; Cr, creatinine; HbA1c, glycatedhaemoglobin; AFP, alphafetoprotein; CEA, carcino-embryonic antigen; CA199, carbohydrate antigen 199; FPG, fasting plasma glucose; FGF21, fibroblast growth factor 21.

**Table 2:** Correlations of FGF21 with other biochemical characteristics in subjects with insulinoma.

Variables	*r*	*P*-value
Age	0.29	0.380
BMI	0.28	0.407
TG	0.36	0.270
ALT	0.66	0.011^*^
AST	0.73	0.011^*^
FPG	0.07	0.850
Fasting C-peptide	0.16	0.631
Fasting insulin	0.80	0.003^**^
Fasting proinsulin	0.72	0.012^*^

^*^P < 0.05, ^**^P < 0.01.

Abbreviations: ALT, alanine aminotransferase; AST, asparatate aminotransferase; TG, triglycerides; FPG, fasting plasma glucose; FGF21, fibroblast growth factor 21.

**Table 3:** Multiple stepwise regression analysis showing the variables independently associated with FGF21.

Independent variable	Standardised β	t	*P*-value
Fasting insulin	0.80	4.00	0.003^**^

^**^P < 0.01.

The original model included age, BMI, fasting insulin, fasting proinsulin, FGF21, ALT, AST.

Abbreviations: ALT, alanine aminotransferase; AST, asparatate aminotransferase; FGF21, fibroblast growth factor 21; TG, triglycerides; BMI, body mass index.
